# Catalpol Attenuates Hepatic Steatosis by Regulating Lipid Metabolism via AMP-Activated Protein Kinase Activation

**DOI:** 10.1155/2020/6708061

**Published:** 2020-04-25

**Authors:** Xiang Tian, Qin Ru, Qi Xiong, Ruojian Wen, Yong Chen

**Affiliations:** ^1^Hubei Province Key Laboratory of Biotechnology of Chinese Traditional Medicine, National & Local Joint Engineering Research Center of High-Throughput Drug Screening Technology, Hubei University, Wuhan Hubei 430062, China; ^2^Wuhan Institute of Biomedical Sciences, School of Medicine, Jianghan University, Wuhan 430056, China

## Abstract

The increased prevalence of nonalcoholic fatty liver disease (NAFLD), which develops from hepatic steatosis, represents a public health challenge. Catalpol, a natural component extracted from the roots of Radix Rehmanniae, has several pharmacological activities. The present study is aimed at examining whether catalpol prevents hepatic steatosis in cell and animal experiments and elucidating the possible mechanisms. HepG2 cells were treated with 300 *μ*M palmitate (PA) and/or catalpol for 24 h in vitro, and male C57BL/6J mice fed a high-fat diet (HFD) were administered catalpol for 18 weeks in vivo. The results revealed that catalpol significantly decreased lipid accumulation in PA-treated HepG2 cells. Moreover, catalpol drastically reduced body weight and lipid accumulation in the liver, whereas it ameliorated hepatocyte steatosis in HFD-fed mice. Notably, catalpol remarkably promoted the phosphorylation of AMP-activated protein kinase (AMPK) and acetyl-CoA carboxylase. Subsequently, catalpol repressed the expressions of lipogenesis-associated genes such as sterol regulatory element-binding protein 1c and fatty acid synthase but promoted the expressions of genes associated with fatty acid *β*-oxidation such as peroxisome proliferator-activated receptor *α* together with its target genes carnitine palmitoyltransferase 1 and acyl-CoA oxidase 1 (ACOX1). However, the preincubation of the HepG2 cells with compound C (10 *μ*M), an AMPK inhibitor, prevented catalpol-mediated beneficial effects. These findings suggest that catalpol ameliorates hepatic steatosis by suppressing lipogenesis and enhancing fatty acid *β*-oxidation in an AMPK-dependent manner. Therefore, catalpol has potential as a novel agent in the treatment of NAFLD.

## 1. Introduction

Nonalcoholic fatty liver disease (NAFLD) is one of the most common causes of hepatic disorders worldwide, and it is strongly associated with obesity, type 2 diabetes mellitus, and dyslipidemia [1–3]. It comprises a wide spectrum of hepatic disorders ranging from simple hepatic steatosis to extremely serious nonalcoholic steatohepatitis, which can develop into fibrosis, cirrhosis, and hepatocellular carcinoma [4]. Studies have demonstrated that the initial symptoms of hepatic steatosis are characterized by triglyceride (TG) overproduction in hepatocytes [5]. Although previously considered to be reversible and relatively benign, it is currently clear that simple steatosis can progress to more extremely hepatic injury if not treated in time [6]. Therefore, it is important to identify strategies to alleviate hepatic steatosis for treating hepatic disorders, specifically associated with NAFLD. Currently, to the best of our knowledge, there is no safe and effective treatment for NAFLD. Lifestyle interventions such as dietary caloric restriction and exercise, which are similar to the recommendations for treating obesity, remain the only approved therapeutic options for this disease [7]. Given the lack of pharmaceutical options, effective and safe therapeutic treatments are urgently needed for managing NAFLD.

Hepatic steatosis develops during the basic progression from early stage to late stage of NAFLD. It is considered a consequence of two major metabolic disorders: increased hepatic lipogenesis and reduced fat consumption [8]. A study has demonstrated that AMP-activated protein kinase (AMPK), a main regulator of cellular energy homeostasis, regulates lipid and glucose metabolism in hepatocytes [9]. Several studies have revealed that AMPK activation could prevent hepatic steatosis by repressing fatty acid synthesis and enhancing fatty acid oxidation. Therefore, AMPK is a conceivably therapeutic target for treating hepatic steatosis [10]. AMPK activation ameliorates hepatic steatosis through multiple mechanisms [11, 12]. Phosphorylated AMPK phosphorylates and inactivates acetyl-CoA carboxylase (ACC), thereby reducing the production of malonyl-CoA, a precursor for fatty acid synthesis that is also a valid inhibitor of carnitine palmitoyltransferase 1 (CPT-1), the rate-limiting enzyme in fatty acid oxidation [13]. Therefore, inhibiting ACC activity reduces lipid synthesis as well as increases fatty acid oxidation. Additionally, sterol regulatory element-binding protein 1c (SREBP-1c), a master transcriptional factor of de novo lipogenesis, is downregulated by AMPK activation, leading to the inhibition of the genes involved in lipogenesis, such as fatty acid synthase (FAS), ACC, and stearoyl-CoA desaturase 1 (SCD1) [14]. Therefore, the AMPK signaling pathway plays crucial roles in reducing lipid accumulation and ameliorating hepatic steatosis.

Several studies have demonstrated that lots of bioactive compounds extracted from traditional Chinese herbs attenuate hepatic lipid accumulation through AMPK activation [15–17]. Catalpol is an iridoid glucoside derived from the roots of the Chinese herb Rehmannia glutinosa. It has remarkable biological activities such as anti-inflammatory, antidiabetic, and neuroprotective properties [18, 19]. Previous research has found that catalpol exerts a protective effect on cholesterol homeostasis in NAFLD by inhibiting endoplasmic reticulum stress and NOX4 overexpression [20]. However, the mechanism by which catalpol improves NAFLD has not been completely elucidated. Therefore, the present study is aimed at investigating whether catalpol ameliorates hepatic steatosis by regulating lipid metabolism in the HepG2 cells treated with palmitate (PA) of mice fed a high-fat diet (HFD). Furthermore, the mechanisms by which PA and HFD promote hepatic steatosis in vitro and in vivo, respectively, were also characterized.

## 2. Materials and Methods

### 2.1. Reagents

Catalpol (98% purity) was purchased from Chengdu Desite Biotechnology Co., Ltd. (Sichuan, China). 3-(4,5-Dimethylthiazol-2-yl)-2,5-diphenyltetrazolium bromide (MTT), free fatty acid-free bovine serum albumin (BSA), PA, Oil Red O, and compound C were purchased from Sigma-Aldrich (St. Louis, MO, USA). Antibodies against SREBP-1c, FAS, and peroxisome proliferator-activated receptor *α* (PPAR*α*) were purchased from Santa Cruz Biotechnology (Santa Cruz, CA, USA). Antibodies against glyceraldehyde 3-phosphate dehydrogenase (GAPDH), AMPK, ACC, p-ACC p-AMPK, and acyl-CoA oxidase 1 (ACOX1) were purchased from Cell Signaling Technology (Beverly, MA, USA). Horseradish peroxidase- (HRP-) conjugated anti-rabbit and anti-mouse secondary antibodies and a bicinchoninic acid (BCA) Protein Assay kit were purchased from Wuhan Boster Biological Engineering Co., Ltd. (Wuhan, China). TG and total cholesterol (TC) kits were purchased from Biosource (USA). Alanine aminotransferase (ALT) and aspartate aminotransferase (AST) kits were purchased from Cusabio (Wuhan, China). All other chemicals used in the present study were of standard analytical grade.

### 2.2. Cell Culture

The human hepatocellular carcinoma cell line HepG2 was purchased from the Typical Culture Preservation Commission Cell Bank, Chinese Academy of Sciences (Shanghai, China). HepG2 cells were maintained in Dulbecco's modified Eagle's medium (Invitrogen, Carlsbad, CA, USA) comprising 10% fetal bovine serum (Invitrogen) and 100 U penicillin/streptomycin (Hyclone, Logan, UT, USA) at 37°C in 5% CO_2_. HepG2 cells were treated with 300 *μ*M PA comprising 1% fat-free BSA in the presence or absence of catalpol for 24 h. Normal group cells were treated with 1% BSA. After treatment, protein expression and TG accumulation were determined according to the purpose of the experiments.

### 2.3. Cell Viability Assay

Briefly, HepG2 cells were seeded at a density of 4 × 10^4^ cells/well into a 96-well plate and incubated overnight. The cells were treated with PA (300 *μ*M) and catalpol (100, 200, or 400 *μ*M) for 24 h. Subsequently, the MTT solution (5 mg/mL) was added to each well and incubated for 4 h. Subsequently, the formed formazan crystals were dissolved in dimethyl sulfoxide after removing the supernatant. The absorbance was measured at 570 nm using a microplate reader (Thermo Scientific, USA). Each treatment was performed in triplicate.

### 2.4. Animal Care and Treatments

A total of 48 male 6-week-old C57BL/6J mice (Vital River Laboratory Animal Technology Co., Ltd., Beijing, China) weighing 23 ± 2 g were included in this study. All of the animals were maintained on a 12 h/12 h light/dark cycle at a temperature of 23°C ± 2°C and 55% ± 5% relative ambient humidity. After acclimation for at least 1 week under laboratory conditions, the experimental mice were randomly and equally divided into the following six groups: normal diet-fed mice (Normal group), HFD-fed mice (HFD group), HFD-fed mice treated with one of the three doses of catalpol (100, 200, or 400 mg/kg), and HFD-fed mice treated with atorvastatin calcium (ATC, 30 mg/kg). The mice in the Normal group were fed a normal diet comprising 10% kcal fat, whereas those in the other five groups were fed an HFD comprising 45% kcal fat. Catalpol and ATC were separately dissolved in saline and administered to the mice via gavage daily for 18 weeks. Body weight was measured once a week. When experiments were terminated, blood and hepatic samples were collected after overnight fasting (12 h) for analysis. The animal protocols were approved by the Animal Care and Use Committee of Jianhan University and performed strictly in compliance with the institutional guidelines for the Care and Use of Laboratory Animals by the National Institutes of Health.

### 2.5. Biochemical Analyses

After overnight fasting (12 h), blood samples were collected and centrifuged (3000 rpm, 15 min, 4°C) to obtain serum for analysis. The serum levels of TG, TC, ALT, and AST were determined using commercially available kits. HepG2 cells were collected after treatment with PA (300 *μ*M) and catalpol (100, 200, or 400 *μ*M) for 24 h. The intracellular accumulation of TG was measured using commercial kits (Biosource). All the experimental kits were used according to the corresponding manufacturers' instructions.

### 2.6. Histological Analysis

Specimens were fixed in 10% formaldehyde solution for 24 h. After fixation, the liver samples were embedded in paraffin. Subsequently, 4 *μ*m thick sections were prepared, deparaffinized with xylene, rehydrated via a series of distilled water and graded ethanol solutions, and stained with hematoxylin and eosin (HE) to investigate histopathological damage in the liver.

### 2.7. Oil Red O Staining

Lipid accumulation in the HepG2 cells in the liver was further evaluated using Oil Red O staining. Briefly, the fixed hepatic tissues were embedded in an optimal cutting temperature compound, and frozen 8 *μ*m sections were prepared using a microtome. HepG2 cells were washed twice with cold phosphate-buffered saline and then fixed in 10% formalin for 30 min. Tissue slices and fixed cell slides were stained with Oil Red O, and oil droplet images were observed using a BX51 microscope (Olympus, Tokyo, Japan) at 200x magnification.

### 2.8. Western Blotting

HepG2 cells or frozen hepatic tissues were homogenized and sonicated in an ice-cold lysis buffer comprising cocktails of protease and phosphatase inhibitors. The homogenates were then centrifuged at 12,000 ×g for 15 min at 4°C to acquire protein for Western blotting analysis. The protein concentration of each sample was measured using a BCA Protein Assay kit. Equal amounts of protein from each group were separated on 10% sodium dodecyl sulfate-polyacrylamide gel electrophoresis gels and transferred into polyvinylidene difluoride membranes (Merck Millipore, MA, USA). Subsequently, the membranes were first blocked in 5% (w/v) nonfat milk for 1 h at room temperature and then immunoblotted with the primary antibodies overnight at 4°C. The membranes were washed three times using Tris-buffered saline comprising Tween-20 (TBST) and then incubated with HRP-conjugated secondary antibody (Boster Company, China) for 1 h at room temperature. The membranes were washed three times with TBST, and an enhanced chemiluminescence reagent (Thermo Fisher, USA) was used to detect the protein bands.

### 2.9. Real-Time Quantitative Polymerase Chain Reaction (RT-qPCR)

Total RNA was isolated from the hepatic tissues of mice using a TRIzol reagent (Invitrogen) according to the standard instructions. One microgram of total RNA was reverse-transcribed to cDNA using an RNeasy kit (QIAGEN). Real-time PCR was performed using Platinum SYBR Green qPCR SuperMix-UDG (Invitrogen) with the following parameters: 94°C for 4 min, 30 cycles of 94°C for 30 s, 56°C for 30 s, 72°C for 25 s, and 72°C for 4 min. GAPDH was used as an endogenous control, and the relative mRNA transcript expressions were determined using the 2^−ΔΔCt^ method. The following primer sequences were used for RT-PCR: SREBP-1c forward, ATGGGTGTGAACCACGAGA; SREBP-1c reverse, CAGGGATGATGTTCTGGGCA; FAS forward, ATCTGGGCTGTCCTGCCTCT; FAS reverse, TTTCACGAACCCGCCTCCTC; GAPDH forward, ATGGGTGTGAACCACGAGA; and GAPDH reverse, CAGGGATGATGTTCTGGGC.

### 2.10. Statistical Analysis

All data are presented as mean ± standard error (SE). Statistical differences between the mean values of different experimental groups were analyzed using a one-way analysis of variance with Tukey's post hoc tests. A *P* value of < 0.05 was considered statistically significant.

## 3. Results

### 3.1. Cytotoxicity of Catalpol and PA in HepG2 Cells

The cytotoxicity of PA and different concentrations of catalpol in HepG2 cells was determined using the MTT assay. No apparent changes in cell viability were detected following treatment with catalpol (100, 200, and 400 *μ*M) and/or 300 *μ*M PA for 24 h. Similar results were also found in cells treated with PA alone.

### 3.2. Catalpol Inhibits Lipid Accumulation in PA-Treated HepG2 Cells

**Figure 1:**
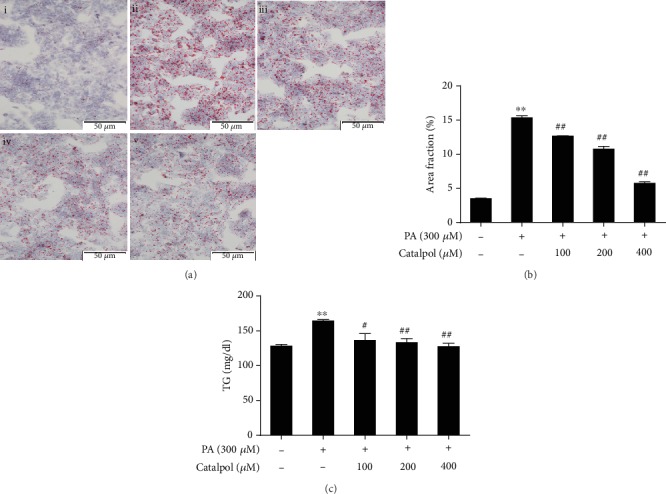
Catalpol inhibits palmitate- (PA-) induced triglyceride (TG) accumulation in HepG2 cells. HepG2 cells were treated with PA (300 *μ*M) and/or catalpol (100, 200, or 400 *μ*M) for 24 h. (a) Lipid accumulation was determined via Oil Red O staining. Images of cells were photographed at 200x magnification: (i) control, (ii) PA (300 *μ*M), (iii) PA (300 *μ*M)+catalpol (100 *μ*M), (iv) PA (300 *μ*M)+catalpol (200 *μ*M), and (v) PA (300 *μ*M)+catalpol (400 *μ*M). (b) A larger red area consisting of lipid droplets in HepG2 cells. (c) Measurement of intracellular TG content. Data are presented as the mean ± SE of three independent experiments. ^∗∗^*P* < 0.01 vs. the Normal group; ^#^*P* < 0.05, ^##^*P* < 0.01 vs. the PA group.

Hepatic steatosis in vitro was induced by treatment with 300 *μ*M PA in HepG2 cells, and the lipid accumulation in cells was determined. Oil Red O staining revealed that treatment with PA induced lipid accumulation, indicating that PA treatment efficiently established a model of hepatic steatosis in vivo. However, treatment with catalpol significantly reduced lipid droplet formation (Figures 1(a) and 1(b)). In addition, enzymatic measurement of the intracellular TG content showed that catalpol significantly decreased lipid accumulation induced by PA (Figure 1(c)).

### 3.3. Catalpol Regulates Enzymes and Genes Involved in Lipid Metabolism in PA-Treated HepG2 Cells

**Figure 2:**
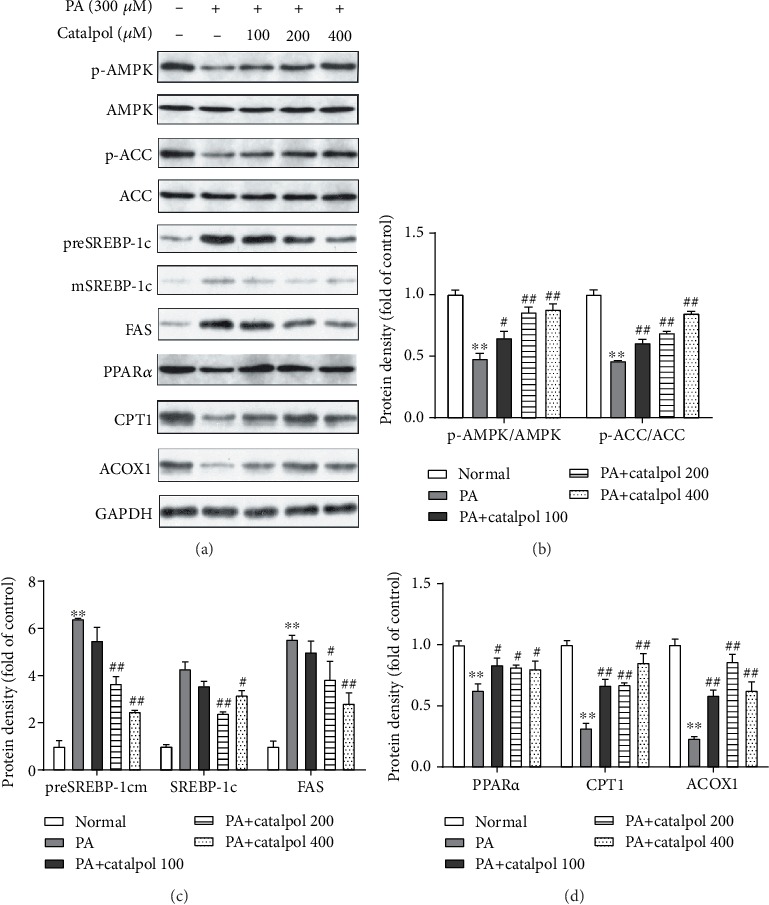
Catalpol treatment regulates enzymes and genes involved in lipid metabolism in palmitate- (PA-) treated HepG2 cells. HepG2 cells were treated with PA (300 *μ*M) and/or catalpol (100, 200, or 400 *μ*M) for 24 h. (a) Protein expressions of p-AMP-activated protein kinase (AMPK), p-acetyl-CoA carboxylase (ACC), precursor and mature sterol regulatory element-binding protein 1c (preSREBP-1c and mSREBP-1c, respectively), fatty acid synthase (FAS), peroxisome proliferator-activated receptor *α* (PPAR*α*), carnitine palmitoyltransferase 1 (CPT1), and acyl-CoA oxidase 1 (ACOX1) were analyzed via Western blotting. (b–d) Densitometric analyses of the band intensity ratios of p-AMPK/AMPK, p-ACC/ACC, preSREBP-1c, mSREBP-1c, FAS, PPAR*α*, CPT1, and ACOX1. Data are presented as the mean ± SE of three independent experiments. ^∗∗^*P* < 0.01 vs. the Normal group; ^#^*P* < 0.05, ^##^*P* < 0.01 vs. the PA group.

**Figure 3:**
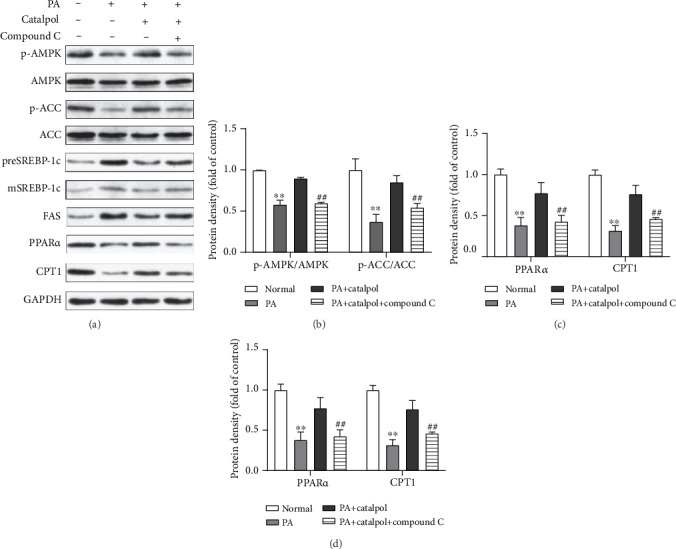
AMPK activation mediates catalpol-regulated lipid metabolism in palmitate- (PA-) treated HepG2 cells. HepG2 cells were treated with PA (300 *μ*M) and/or catalpol (400 *μ*M) for 24 h. Compound C (10 *μ*M) was added 2 h prior to the cotreatment with PA and catalpol. (a) Protein expressions of p-AMP-activated protein kinase (AMPK), p-acetyl-CoA carboxylase (ACC), precursor and mature sterol regulatory element-binding protein 1c (preSREBP-1c and mSREBP-1c, respectively), fatty acid synthase (FAS), peroxisome proliferator-activated receptor *α* (PPAR*α*), and carnitine palmitoyltransferase 1 (CPT1) were analyzed via Western blotting. (b–d) Densitometric analyses of the band intensity ratios of p-AMPK/AMPK, p-ACC/ACC, preSREBP-1c, mSREBP-1c, FAS, PPAR*α*, and CPT1. Data are presented as the mean ± SE of three independent experiments. ^∗∗^*P* < 0.01 vs. the Normal group; ^#^*P* < 0.05, ^##^*P* < 0.01 vs. the catalpol group.

To clarify the mechanisms underlying the beneficial effects of catalpol on lipid accumulation induced by PA, we analyzed the lipogenesis genes and fatty acid oxidation genes in HepG2 cells. Figures 2(a) and 2(b) reveal that PA treatment markedly decreased the phosphorylation of AMPK and ACC in HepG2 cells. However, catalpol treatment efficiently enhanced their phosphorylation in a concentration-dependent manner. Subsequently, we found that PA treatment significantly increased the protein expressions of both precursor and mature SREBP-1c and FAS in HepG2 cells, whereas catalpol treatment significantly reversed these PA-induced effects (Figures 2(a) and 2(c)). Next, we examined the expression of proteins involved in fatty acid *β*-oxidation in PA-treated HepG2 cells. Data presented in Figures 2(a) and 2(d) illustrated that PA treatment greatly decreased the protein expression of PPAR*α* and its target genes including CPT1 and ACOX1, whereas catalpol administration significantly increased their protein expressions. Furthermore, we evaluated whether AMPK activation mediated the inhibitory effect of catalpol on lipid metabolism. HepG2 cells were pretreated with the AMPK inhibitor compound C for 2 h prior to treatment with PA and catalpol. As shown in Figure 3, pretreatment with compound C blocked the effects of catalpol treatment on the phosphorylation of AMPK and ACC in PA-treated HepG2 cells (Figures 3(a) and 3(b)). Moreover, compound C abolished the inhibitory effect of catalpol on the expressions of both precursor and mature SREBP-1c and FAS (Figures 3(a) and 3(c)). Similarly, compound C also blocked the enhancement of PPAR*α* and CPT1 treated by catalpol in PA-treated HepG2 cells (Figures 3(a) and 3(d)). Taken together, these results demonstrated that catalpol inhibited lipogenesis and stimulated fatty acid *β*-oxidation through the AMPK pathway, thereby inhibiting intracellular TG accumulation in HepG2 cells.

### 3.4. Catalpol Treatment Reduces Body Weight and Elevates Serum Levels of Lipids and Hepatic Enzymes in HFD-Fed Mice

**Figure 4:**
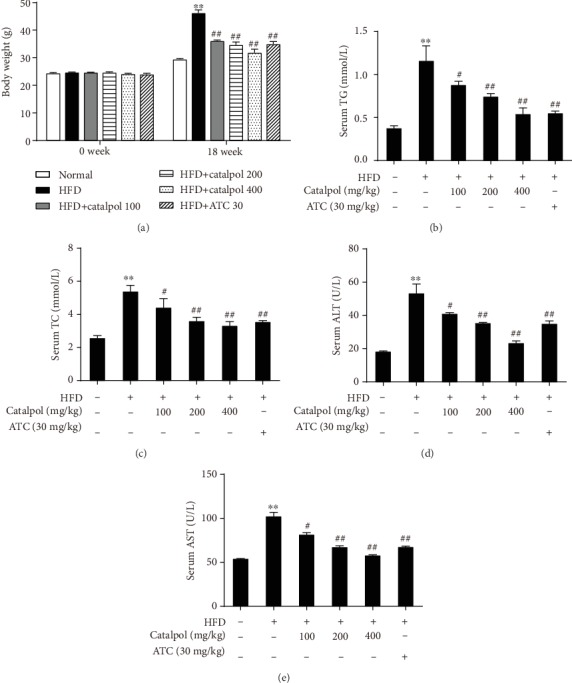
Catalpol treatment reduces body weight gain and elevates the serum levels of lipids and hepatic enzymes in high-fat diet- (HFD-) fed mice. C57BL/6J mice were fed a normal diet or HFD and treated with saline, atorvastatin calcium (ATC), or different doses of catalpol daily for 18 weeks. (a) Body weight changes. (b–e) Serum levels of triglyceride (TG), total cholesterol (TC), alanine aminotransferase (ALT), and aspartate aminotransferase (AST). Data are presented as the mean ± SE (n = 8). ^∗∗^*P* < 0.01 vs. the Normal group; ^#^*P* < 0.05, ^##^*P* < 0.01 vs. the HFD group.

Catalpol (100, 200, or 400 mg/kg) was administered daily to mice for 18 weeks to investigate its effects on hepatic steatosis. The initial body weight of the mice was not remarkably different among the groups. After 18 weeks, the body weight of HFD-fed mice was significantly higher than that of mice fed a normal diet. However, catalpol supplementation significantly decreased the body weight gain induced by HFD feeding in a dose-dependent manner (Figure 4(a)). Subsequently, fasting serum biochemical indicators were examined. Figures 4(b) and 4(c) present remarkable increases in the serum levels of TG and TC in HFD-fed mice compared with those in mice fed a normal diet. Catalpol administration significantly decreased the serum levels of TG and TC in a dose-dependent manner compared with those observed in HFD-fed mice. Additionally, HFD feeding also resulted in elevated serum levels of ALT and AST in HFD-fed mice compared with those observed in the Normal group. However, catalpol treatment significantly blocked the elevation of the serum levels of ALT and AST in a dose-dependent manner compared with that in the HFD group (Figures 4(d) and 4(e)).

### 3.5. Catalpol Treatment Ameliorates Hepatic Steatosis in HFD-Fed Mice

**Figure 5:**
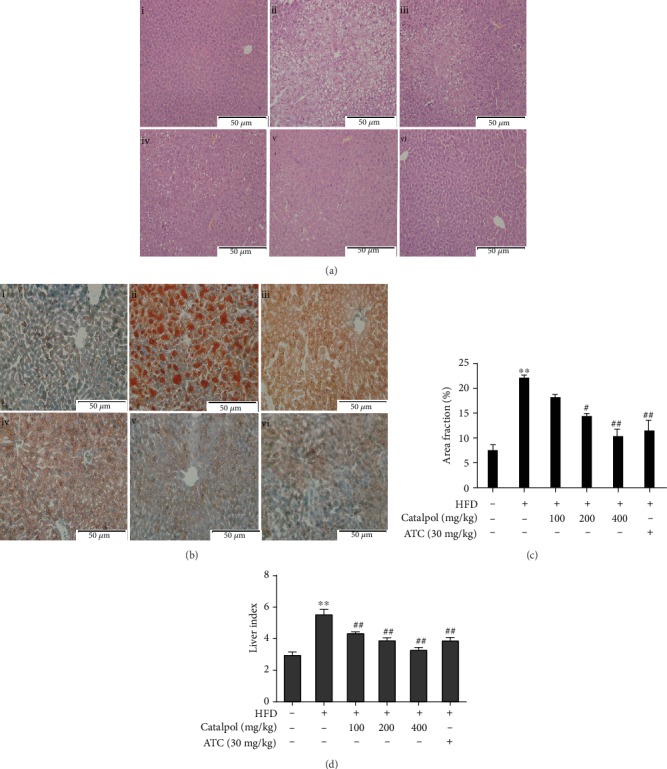
Catalpol treatment prevented hepatic steatosis in high-fat diet- (HFD-) fed mice. (a) Hematoxylin and eosin staining of the hepatic sections (200x): (i) normal, (ii) HFD, (iii) HFD+catalpol (100 mg/kg, oral gavage), (iv) HFD+catalpol (200 mg/kg, oral gavage), (v) HFD+catalpol (400 mg/kg, oral gavage), and (vi) HFD+atorvastatin calcium (ATC, 30 mg/kg, oral gavage). (b) Oil Red O staining of the hepatic sections (200x): (i) normal, (ii) HFD, (iii) HFD+catalpol (100 mg/kg, oral gavage), (iv) HFD+catalpol (200 mg/kg, oral gavage), (v) HFD+catalpol (400 mg/kg, oral gavage), and (vi) HFD+ATC (30 mg/kg, oral gavage). (c) Percentage area of hepatic tissue occupied by lipid droplets. (d) Liver index. Data are presented as the mean ± SE (n = 8). ^∗∗^*P* < 0.01 vs. the Normal group; ^#^*P* < 0.05, ^##^*P* < 0.01 vs. the HFD group.

To investigate whether catalpol treatment ameliorated hepatic steatosis in HFD-fed mice, hepatic tissues were assessed via HE and Oil Red O staining using light microscopy as well as digital image analysis (DIA). Increased microvesicular steatosis was observed in HE-stained sections of hepatocytes from HFD-fed mice compared with the findings in mice fed a normal diet, whereas steatosis was obviously lessened in hepatocytes from mice treated with catalpol (Figure 5(a)). Consistent with the HE staining results, Oil Red O staining revealed that lipid droplet levels were significantly increased in hepatocytes from HFD-fed mice, whereas hepatic lipid droplet accumulation was significantly ameliorated by treatment with catalpol (Figure 5(b)). The DIA results revealed obvious decreases in lipid droplet levels in mice treated with catalpol (Figure 5(c)). In agreement with the histological data, the liver index, representing the relative liver weight, was significantly higher in HFD-fed mice than in normal diet-fed mice, whereas catalpol administration reduced the relative liver weight in a dose-dependent manner (Figure 5(d)).

### 3.6. Catalpol Treatment Regulates Genes Involved in Hepatic Lipid Metabolism in HFD-Fed Mice

**Figure 6:**
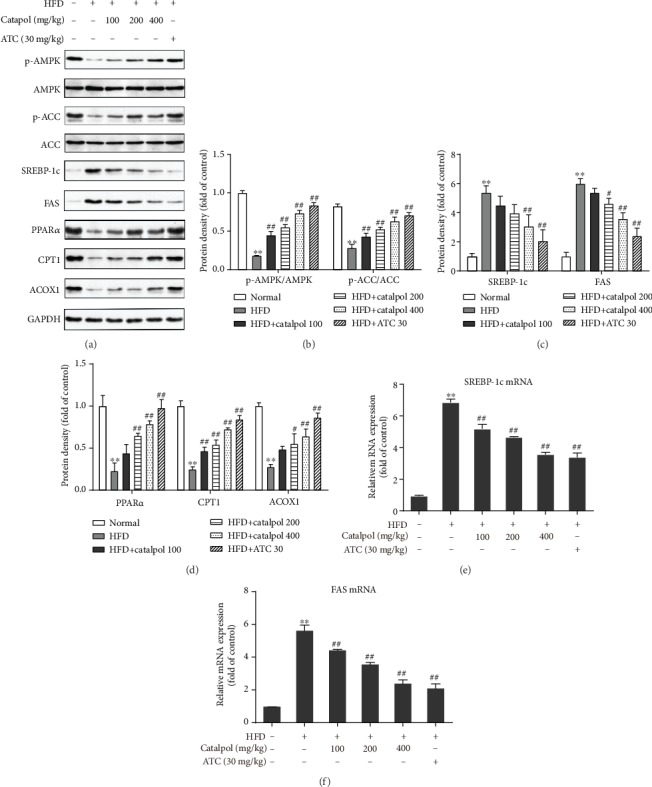
Catalpol treatment modulated genes involved in hepatic lipid metabolism in the livers of high-fat diet- (HFD-) fed mice. (a) Protein expressions of p-AMP-activated protein kinase (AMPK), p-acetyl-CoA carboxylase (ACC), sterol regulatory element-binding protein 1c (SREBP-1c), fatty acid synthase (FAS), peroxisome proliferator-activated receptor *α* (PPAR*α*), carnitine palmitoyltransferase 1 (CPT1), and acyl-CoA oxidase 1 (ACOX1) were analyzed via Western blotting. (b–e) Densitometric analyses of the band intensity ratios of p-AMPK/AMPK, p-ACC/ACC, SREBP-1c, FAS, PPAR*α*, CPT1, and ACOX1. (e, f) Relative mRNA expression of SREBP-1c and FAS. Data are presented as the mean ± SE of three independent experiments. ^∗∗^*P* < 0.01 vs. the Normal group; ^#^*P* < 0.05, ^##^*P* < 0.01 vs. the HFD group.

To confirm the mechanisms of the catalpol-mediated amelioration of hepatic steatosis, the expressions of hepatic genes involved in lipogenesis and fatty acid *β*-oxidation were evaluated in vivo. In agreement with the in vitro results, AMPK and ACC phosphorylation was decreased in the hepatocytes of HFD-fed mice, and this finding was substantially reversed by catalpol treatment (Figures 6(a) and 6(b)). We then evaluated the genes involved in lipogenesis. The protein expressions of SREBP-1c and FAS in the livers of HFD-fed mice were markedly decreased by catalpol administration (Figures 6(a) and 6(c)). The mRNA expressions of SREBP-1c and FAS were further determined by RT-qPCR, and consistent with the protein expression results, catalpol reversed the HFD-mediated increases in SREBP-1c and FAS mRNA expression (Figures 6(e) and 6(f)). Moreover, catalpol treatment in HFD-fed mice resulted in significant increases in the protein expressions of PPAR*α*, CPT1, and ACOX1, which are involved in fatty acid *β*-oxidation (Figures 6(a) and 6(d)).

## 4. Discussion

NAFLD is considered a risk factor for various metabolic disorders such as obesity, diabetes, and dyslipidemia, making it a growing public health concern worldwide [21]. Hepatic steatosis develops in the earliest stage of NAFLD, and it is also benign in this stage. However, it can evolve into more severe hepatic damage. Therefore, it is important to control NAFLD at the initial stage. Previous research has recently reported that plant extracts or high-purity compounds could attenuate hepatic steatosis and eventually improve NAFLD by stimulating the AMPK pathway in hepatocytes [22–24]. The present study investigated the preventive effect of catalpol on hepatic steatosis and elucidated its underlying mechanisms in PA-treated HepG2 cells and HFD-fed obese mice. The results of our study demonstrate that catalpol treatment effectively decreases hepatic lipid accumulation in cell and animal experiments probably through the activation of the AMPK signaling pathway and subsequent suppression of lipogenesis and enhancement of fatty acid *β*-oxidation, suggesting that catalpol exerted protective effects against hepatic steatosis and thus prevented the development of NAFLD.

Although the pathogenesis of NAFLD is unclear, significant evidence indicates that NAFLD is closely associated with lipid accumulation and the dysfunction of lipid metabolism [25]. Any of the mechanisms involved in hepatic lipid accumulation could represent useful targets to block the development of NAFLD. In particular, hepatic steatosis is the initial step in the development of NAFLD, and it occurs when the homeostasis between TG synthesis and clearance is destructed [26]. Meanwhile, AMPK is a primary regulator of several metabolic pathways, and the activation of AMPK inhibits hepatic steatosis through several mechanisms [11, 12, 27]. First, AMPK inhibits lipogenesis by inactivating ACC and SREBP-1c. ACC is a crucial lipogenic enzyme that blocks fatty acid oxidation and initiates de novo lipogenesis by increasing the production of malonyl-CoA, a precursor for fatty acid synthesis. After activation, AMPK phosphorylates ACC and attenuates its activity [28]. In addition, SREBP-1c is the master transcription factor regulating lipid synthesis in hepatocytes, which is conducive to the pathogenesis of hepatic steatosis [29]. Activation of SREBP-1c during lipid accumulation appears to be essential for the development of hepatic steatosis [30]. Several studies have demonstrated that SREBP-1c is upregulated in the livers of patients and mice with hepatic steatosis [31–33]. Additionally, the suppression of SREBP-1c induces the downregulation of ACC and FAS, which are two key enzymes regulating lipid homeostasis [34]. FAS can catalyze the de novo synthesis of fatty acids, whereas ACC is the rate-limiting enzyme in fatty acid synthesis. The transcriptional activity of SREBP-1c is negatively regulated by the activation of AMPK [33, 35]. Previous studies have reported that several biologically active compounds purified from Chinese medical herbs could ameliorate lipid accumulation by activating AMPK signaling and the subsequent reduction in SREBP-1c expression in hepatocytes [16, 17, 36]. Therefore, we inferred the involvement of the AMPK/SREBP-1c signaling pathway in the attenuation of hepatic lipid accumulation observed in the present study. As hypothesized, pretreatment with compound C prevented these catalpol-mediated effects. Our results suggested that catalpol exerts preventive effects on hepatic lipid accumulation possibly by inducing AMPK activation and subsequently suppressing SREBP-1c-mediated lipogenesis.

Second, in addition to suppressing lipogenesis in the liver during lipid metabolism, AMPK also induces fatty acid *β*-oxidation by upregulating the PPAR*α*-regulated fatty acid oxidation genes, including CPT1 and ACOX1 [11]. Fatty acid oxidation is another metabolic pathway that maintains hepatic lipid homeostasis, and impaired fatty acid oxidation leads to abnormal hepatic accumulation of TGs and results in hepatic steatosis [37, 38]. PPAR*α* is a master regulator of the genes involved in fatty acid oxidation, and it blocks hepatic lipid deposition by promoting fatty acid catabolism [39–41]. CPT1 and ACOX1, the target genes of PPAR*α*, are the rate-limiting enzymes of fatty acid oxidation, and they are used as indices of mitochondrial oxidation activity [42]. The findings of our study demonstrated that catalpol treatment enhanced fatty acid oxidation by activating PPAR*α*-mediated gene transcription and improving CPT1 and ACOX1 activity, thereby attenuating hepatic steatosis. However, pretreatment with compound C prevented these catalpol-mediated effects.

The protective effects of catalpol on hepatic steatosis were further verified in vivo. HFD-fed mice represent a useful model for studying NAFLD [43]. Therefore, an HFD-fed mouse model was established in our study. Our findings of reduced serum levels of TG, TC, ALT, and AST suggest that catalpol significantly ameliorates hepatic damage. Consistent with the results of cell experiments, the results of Oil Red O staining indicated that catalpol restores hepatic function and regulates lipid metabolism in HFD-fed mice. Furthermore, in line with the in vitro data, catalpol reversed HFD-mediated reductions of AMPK phosphorylation. We also observed that catalpol treatment decreased the expressions of lipogenesis genes (SREBP-1c, FAS, and ACC) and increased the expressions of fatty acid oxidation genes (PPAR*α*, CPT1, and ACOX1).

## 5. Conclusions

In the present study, we confirmed that catalpol is effective against hepatic steatosis in a PA-induced cell model and an HFD-induced mouse model. AMPK activation might contribute to the subsequent inhibition of SREBP-1c-mediated lipogenesis and the enhancement of PPAR*α*-mediated fatty acid *β*-oxidation. This evidence supports the potential utility of catalpol as a novel agent for treating hepatic steatosis and decreasing the risk of NAFLD.

## Data Availability

The data used to support the findings of this study are included within the article.
