# Correction to “Catalpol Attenuates Hepatic Steatosis by Regulating Lipid Metabolism via AMP‐Activated Protein Kinase Activation”

**DOI:** 10.1155/bmri/9760936

**Published:** 2025-11-04

X. Tian, Q. Ru, Q. Xiong, R. Wen, Y. Chen, “Catalpol Attenuates Hepatic Steatosis by Regulating Lipid Metabolism via AMP‐Activated Protein Kinase Activation,” *BioMed Research International*, 2020, https://doi.org/10.1155/2020/6708061

In the article, there is an error in Figure 3c, which was mistakenly duplicated from Figure 3d in the production process. The correct Figure 3 is shown as follows:

Figure 3AMPK activation mediates catalpol‐regulated lipid metabolism in palmitate (PA)‐treated HepG2 cells. HepG2 cells were treated with PA (300 *μ*M) and/or catalpol (400 *μ*M) for 24 h. Compound C (10 *μ*M) was added 2 h prior to the cotreatment with PA and catalpol. (a) Protein expressions of *p*‐AMP‐activated protein kinase (AMPK), *p*‐acetyl‐CoA carboxylase (ACC), precursor and maturesterol regulatory element‐binding Protein 1c (preSREBP‐1c and mSREBP‐1c, respectively), fatty acid synthase (FAS), peroxisome proliferator–activated receptor *α* (PPAR*α*), and Carnitine Palmitoyltransferase 1 (CPT1) were analyzed via western blotting. (b–d) Densitometric analyses of the band intensity ratios of *p*‐AMPK/AMPK, *p*‐ACC/ACC, preSREBP‐1c, mSREBP‐1c, FAS, PPAR*α*, and CPT1. Data are presented as the mean ± SE of three independent experiments. ∗∗*p* < 0.01 versus the normal group; #*p* < 0.05, ##*p* < 0.01 versus the catalpol group.

**Figure:**
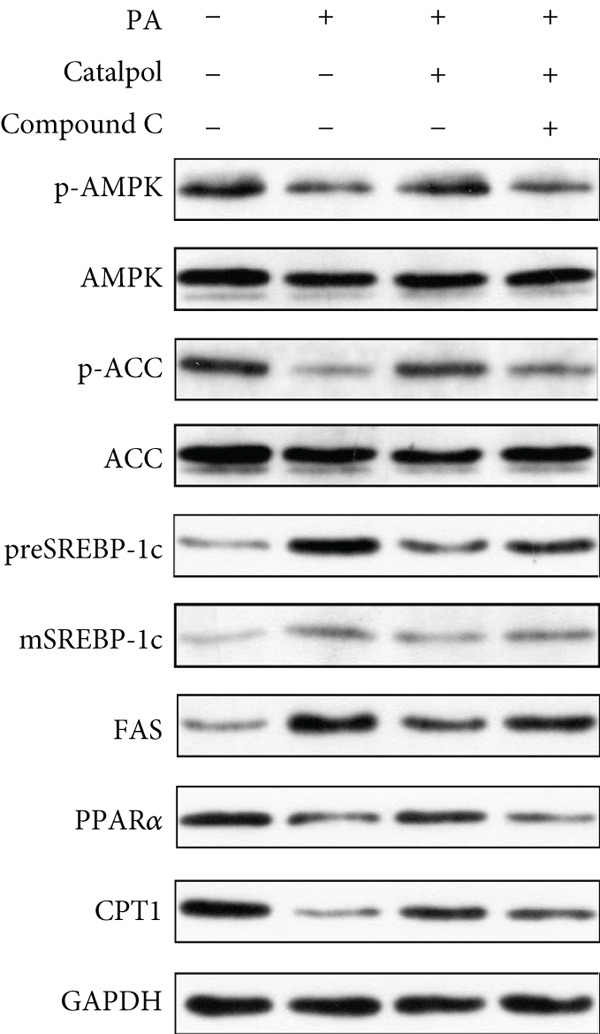
(a)

**Figure:**
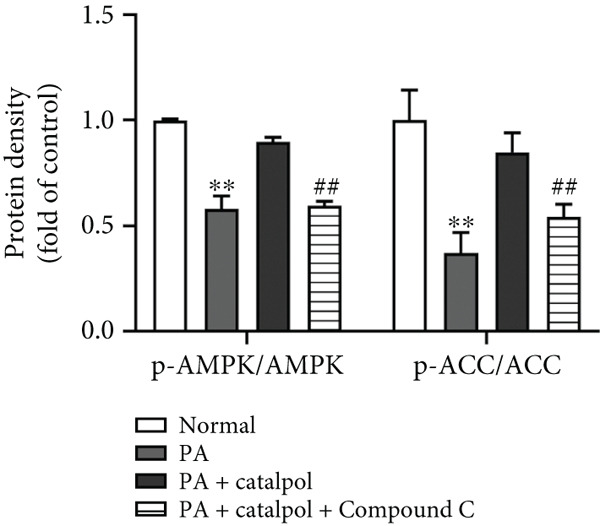
(b)

**Figure:**
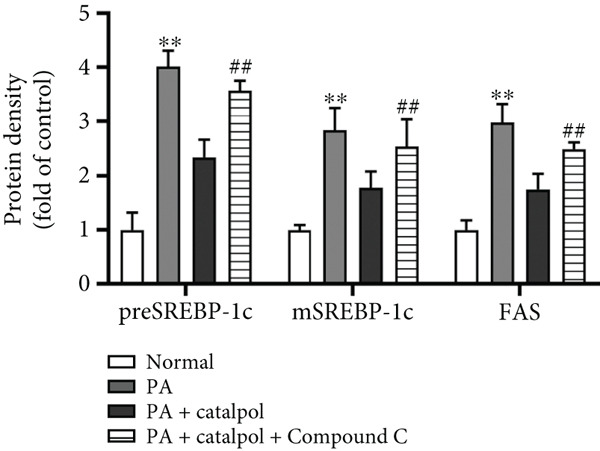
(c)

**Figure:**
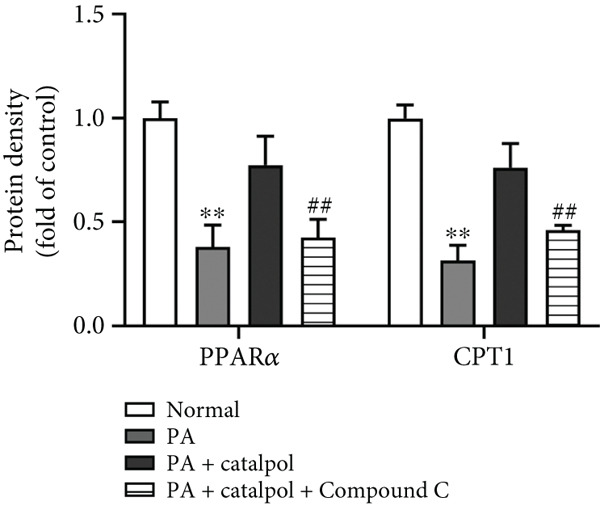
(d)

We apologize for this error.

